# Diagnostic challenges in infant death associated with maternal cocaine abuse during pregnancy and lactation: a forensic case report and review of the literature

**DOI:** 10.3389/fped.2026.1854801

**Published:** 2026-08-26

**Authors:** Naomi Iacoponi, Rebecca Maggi, Vincenzo Nardini, Silvio Chericoni, Emanuela Turillazzi, Marco Di Paolo

**Affiliations:** 1 Department of Public Health and Infectious Diseases, Sapienza University of Rome, Rome, Italy; 2 Department of Surgical, Medical, and Molecular Pathology and Critical Care Medicine, Institute of Legal Medicine, University of Pisa, Pisa, Italy; 3 Department of Oncology, Second Division of Pathology, Pisa University Hospital, Pisa, Italy

**Keywords:** forensic, infant cocaine exposure, maternal cocaine abuse, pediatric pathology, SIDS

## Abstract

**Introduction:**

Maternal cocaine abuse during pregnancy and lactation represents a critical public health crisis. Recent 2024-2025 data indicate approximately 4.3 million users in the United States and 2.7 million young adults in Europe. While epidemiological evidence suggests an independent association between prenatal cocaine exposure and Sudden Infant Death Syndrome (SIDS), forensic interpretation remains complex due to the lack of validated toxicological thresholds for the pediatric population.

**Method:**

We report the case of a 36-day-old infant who died suddenly and unexpectedly. Post-mortem investigations were performed to differentiate between SIDS and toxicological intoxication. Forensic hair analysis confirmed chronic cocaine exposure, occurring both in utero and via lactation. A systematic review of the literature was conducted in accordance with the PRISMA (Preferred Reporting Items for Systematic Reviews and Meta-Analyses) guidelines. The search focused on fetal and infant fatalities associated with maternal cocaine consumption to identify recurrent toxicological patterns.

**Results:**

The systematic review identified seven key studies describing neonatal deaths linked to maternal cocaine use. A significant discrepancy was observed between adult post-mortem concentrations, ranging from 900 ng/mL in cardiac-related deaths to over 19,000 ng/mL in acute intoxications, and neonatal levels, which showed mean concentrations of 39 ng/mL, though extreme cases have reached 4.8 mg/dL. This variability underscores the difficulty in establishing a lethal range for infants.

**Conclusion:**

The findings highlight a critical gap in forensic pathology and toxicology. There is an urgent need for systematic data collection during autopsies to establish standardized reference intervals. Improving these datasets is essential for forensic pathologists to accurately distinguish between incidental exposure and cocaine-induced fatality in pediatric cases.

## 1 Introduction

Cocaine abuse during pregnancy and lactation is a significant and persistent global public health problem. In 2024, the Substance Abuse and Mental Health Service Administration estimated that 73.6 million people aged 12 or older used illicit drugs, of which 4.3 millions used cocaine (1). In Europe, cocaine was the second most used illicit drug in 2025, with almost 2.7 million aged 15- to 34-year-old reporting use (2). Women account for approximately one quarter of those with severe illicit drug problems, and those who use drugs often experience greater stigma than men because they are perceived as violating expected caregiving and maternal roles; this stigma, coupled with unsupportive or discriminatory services, can intensify guilt and shame and deter help-seeking. Cocaine use during pregnancy is associated with a wide range of adverse obstetric and neonatal outcomes, including low birth weight, prematurity, respiratory distress, genitourinary malformations, cerebral and bowel infarctions, reduced head circumference, and an increased risk of seizures (3).

Prenatal cocaine exposure has also been linked to a higher risk of sudden infant death syndrome (SIDS), although the evidence is not fully consistent (4–6). A large meta-analysis including millions of infants demonstrated a significantly increased risk of SIDS among those prenatally exposed to substances, independent of socioeconomic factors. Similarly, population-based data have shown higher SIDS rates among drug-exposed infants compared with non-exposed infants, although the risk appears greater with opioid exposure than with cocaine (7).

Overall, while the incidence of SIDS has declined over time, it remains consistently higher among infants with prenatal drug exposure (8). In this context, the identification of cocaine-related deaths represents a critical challenge for the forensic pathologist. This task is particularly complex in the pediatric population, as reliable reference concentrations for diagnosing cocaine overdose are lacking, especially in infants.

In adults, postmortem toxicological data provide more robust reference ranges. Analysis of 5,339 cases reported to the National Programme on Substance Abuse Deaths (2000–2019) showed that mean blood cocaine concentrations were approximately 900 ng/mL in cardiac-related deaths, compared with markedly higher levels in acute intoxication (≈19,100 ng/mL) and chronic or binge use (≈6,200 ng/mL) (9). However, significant variability exists, and exceptionally high concentrations have been reported, such as a fatal case with a postmortem blood level of 104 mg/L (10). Additional case series confirm the heterogeneity of cocaine-related deaths in adults, with both fatal intoxications and non–drug-related deaths occurring at overlapping blood concentrations. Cocaine and its metabolites (particularly benzoylecgonine) are frequently detected even in cases not directly attributable to acute toxicity, further complicating interpretation (11).

By contrast, in neonates and infants, available toxicological data are extremely limited, and concentration thresholds associated with clinical toxicity or fatal outcomes remain undefined. This lack of reference values significantly hampers the interpretation of postmortem findings and the attribution of cause of death in suspected cocaine exposure, underscoring the need for detailed case documentation and systematic data collection to improve diagnostic accuracy in this vulnerable population.

In this context, we present the case of a 36-day-old infant with documented prenatal and postnatal cocaine exposure. The medicolegal investigation highlights the diagnostic difficulties in attributing a definitive cause of death, given the substantial knowledge gap regarding lethal concentrations in infants.

## 2 Materials and methods

### 2.1 Study design and PICOS framework

We conducted a systematic review in accordance with the Preferred Reporting Items for Systematic Reviews and Meta-Analyses (PRISMA) 2009 guidelines. The review question was structured using the PICOS framework. The population comprised fetuses and infants who were exposed to maternal cocaine use during pregnancy, lactation, or both. The exposure of interest was maternal cocaine consumption, as documented by toxicological analysis or clinical history. The eligible study designs included original research articles, case reports, case series, and reviews. Because the available literature consisted predominantly of observational and case-based reports, formal matched unexposed comparator cohorts were absent in most included studies; this was considered a key limitation of the evidence base. The primary outcomes were defined *a priori* as: (1) infant death attributed to acute cocaine toxicity, based on toxicological and pathological findings supporting direct causation; and (2) unexplained sudden infant death, including sudden unexpected infant death (SUID) and sudden infant death syndrome (SIDS), in the setting of documented cocaine exposure without conclusive evidence of acute intoxication. This distinction was maintained throughout data extraction, analysis, and synthesis to avoid conflating toxicological causation with associative exposure findings.

### 2.2 Data extraction

Two authors (N.I. and R.M.) independently extracted data from each included study using a standardized extraction template. The template captured the following variables: author(s), year of publication, country of origin, study design, sample size, gestational age or postnatal age at death, route and duration of maternal cocaine exposure, biological matrix tested, analytical method employed, cocaine and benzoylecgonine concentrations, cause of death (when reported), and relevant pathological findings at autopsy. Discrepancies between reviewers were resolved by consensus; when agreement could not be reached, a third reviewer (V.N.) provided arbitration. Data were synthesized narratively because the heterogeneity across studies precluded quantitative pooling.

### 2.3 Risk of bias assessment

The methodological quality of each included study was assessed independently by two authors (E.T. and M.D.P.). Each study was rated on domains including selection, comparability, and outcome assessment. The overall quality of the evidence was judged to be low to moderate, driven primarily by small sample sizes, retrospective designs, and the absence of standardized analytical protocols across laboratories.

### 2.4 Reasons for excluding meta-analysis

A formal meta-analysis was not performed due to substantial inter-study heterogeneity in the following domains: analytical platforms (gas chromatography-mass spectrometry, immunoassay, and liquid chromatography-tandem mass spectrometry), biological matrices (venous blood, umbilical cord blood, meconium, and hair), and the heterogeneous composition of the study populations with respect to gestational age, exposure patterns, and geographical context. The limited number of eligible studies (*n* = 7) further precluded meaningful pooled analysis or reliable assessment of publication bias using funnel plots or statistical tests such as Egger’s test. This should be considered a limitation of the review.

## 3 Results

### 3.1 Review of the literature

**Figure 1:**
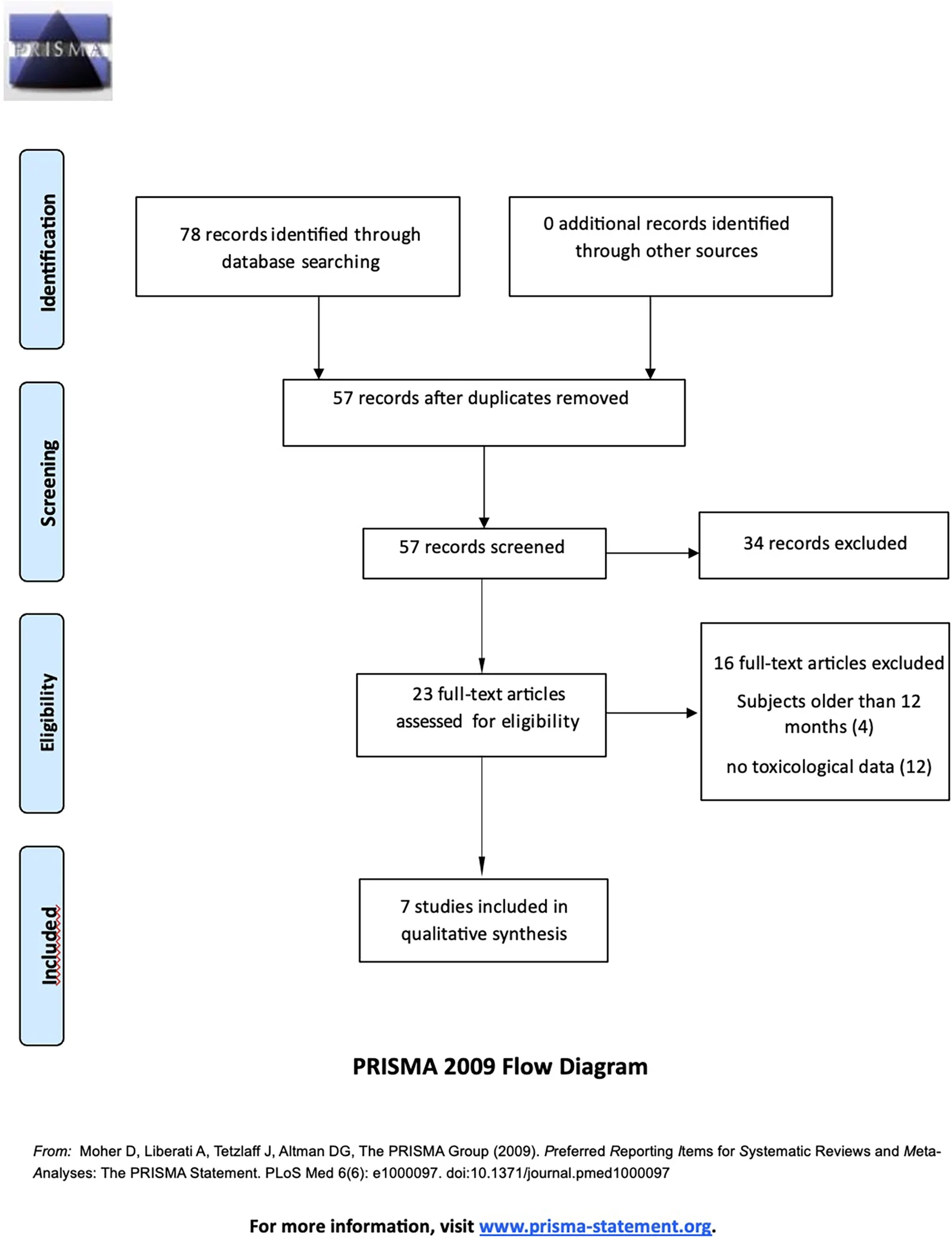
This figure shows our search strategy according to PRISMA 2009 standards.

The PRISMA-compliant search identified 78 records across the four databases. After removal of duplicates, 57 records were screened by title and abstract, yielding 23 articles for full-text assessment. Of these, 7 studies met the inclusion criteria and were included in the final review. The reasons for exclusion at the full-text stage were: non-English language (*n* = 4), toxicological levels not reported (*n* = 6), age of exposed children exceeding 1 year (*n* = 3), and insufficient clinical or pathological detail (*n* = 3). The PRISMA flow diagram with numerical counts at each stage and categorized exclusion reasons is presented in Figure 1. Among the seven included studies, cases were heterogeneous with respect to the assigned cause of death. In a subset of cases, autopsy and toxicological findings supported a diagnosis of acute cocaine intoxication, with detectable cocaine and benzoylecgonine in blood and, in some cases, in brain and liver tissue. In other cases, cocaine exposure was unequivocally documented by toxicological analysis, yet the pathological findings were nonspecific and consistent with SUID or SIDS, including subepicardial petechiae, pulmonary hemorrhages, and arcuate nucleus hypoplasia. This overlap underscores the absence of validated toxicological thresholds that reliably distinguish fatal cocaine intoxication from incidental exposure in infants. Across the review, similar or overlapping cocaine and benzoylecgonine concentration ranges were observed in both fatal and non-fatal cases, further complicating attribution of cause of death.

Figure 1 illustrates the search strategy according to the PRISMA 2009 method.

Table 1 summarizes the findings of the systematic review, which identified seven key studies investigating fetal and infant deaths linked to maternal cocaine use. Collectively, these reports encompass a cohort of 421 subjects, including 92 documented deaths with confirmed cocaine exposure, 289 infants screened *in utero* or during lactation, and one case of accidental postnatal ingestion. Toxicological analyses revealed a substantial spectrum of cocaine concentrations across different matrices. In fetal deaths, Meeker et al. reported average blood cocaine and benzoylecgonine levels of 0.26 mg/L and 1.73 mg/L, respectively. In individual infant fatalities, blood concentrations ranged from 26 µg/L to 94 µg/L, with corresponding brain and liver levels of 19–40 µg/L and 24–122 µg/L. Conversely, screening of umbilical cord plasma and urine in neonates showed a markedly lower mean concentration of 39 ng/mL. In a larger post-mortem cohort of 64 infants, concentrations reached a peak of 4.8 mg/L, illustrating extreme toxicological variance. These data demonstrate a significant overlap between cocaine levels in fatal and non-fatal cases. Such findings underscore the current lack of definitive toxicological thresholds and highlight the challenges faced by forensic pathologists in attributing infant mortality exclusively to cocaine toxicity in the absence of pathognomonic findings.

**Table 1:** Summarizes the results of our revision of the literature.

Authors	Number of cases	Brief case description	Toxicological findings
Meeker et al. (44)	25	Fetal and newborn death associated with maternal cocaine use	Average fetal blood cocaine was 260 ng/mL, benzoylecgonine was 1,730 ng/mL
Basilicata et al. (45)	1	8-month-old baby girl found dead in the cradle	Cocaine in blood 600 ng/mL, in Liver 0,10 ng/mg Brain 0.13 ng/mg Benzoylecgonine 0.28 ng/mL 0.12 ng/mg, and 0.10 ng/mg
Cravey (46)	2	8- and 10-week-old infants’ deaths attributed to cocaine intoxication	Case n.1 8-week-old 26 ng/mL of cocaine in blood, 19 ng/mL in brain, 24 ng/mL in liver Case n.2 10-week-old 94 ng/mL in blood, 40 ng/mL in brain, 122 ng/mL in liver
Chiriboga et al. (47)	253	Infants alive were evaluated 1–7 days after birth	Low exposure 2–66 ng/mg high exposure 81–4,457 ng/mg
Dempsey et al. (21)	36	Alive neonates at risk for prenatal cocaine exposure	Umbilical cord plasma and urine were examined, 9 infants tested positive for cocaine (max concentration 88 ng/mL, mean 39 ng/mL)
Morild et al. (48)	103	Toxicologic screening for cocaine and its metabolites was performed on 103 cases of fetal death	64 infants tested positive for cocaine. The highest blood concentration found was 4,800 ng/mL, while the mean concentration was 800 ng/mL.
Garland et al. (53)	1	A case of a 9-month-old male who accidentally ingested cocaine, the baby reported no sequele and was discharged from hospital	The serum cocaine level was 190 mg/dL, and the benzoylecgonine level was 2,360 ng/mL.

It compiles toxicological findings from seven studies published between 1988 and 2024, detailing fetal and infant exposure to cocaine. The table encompasses over 400 cases in which toxicological analysis was performed on various biological matrices.

ng/mL, nanograms per milliliter; ng/mg, nanograms per milligrams.

### 3.2 Case presentation

**Figure 2:**
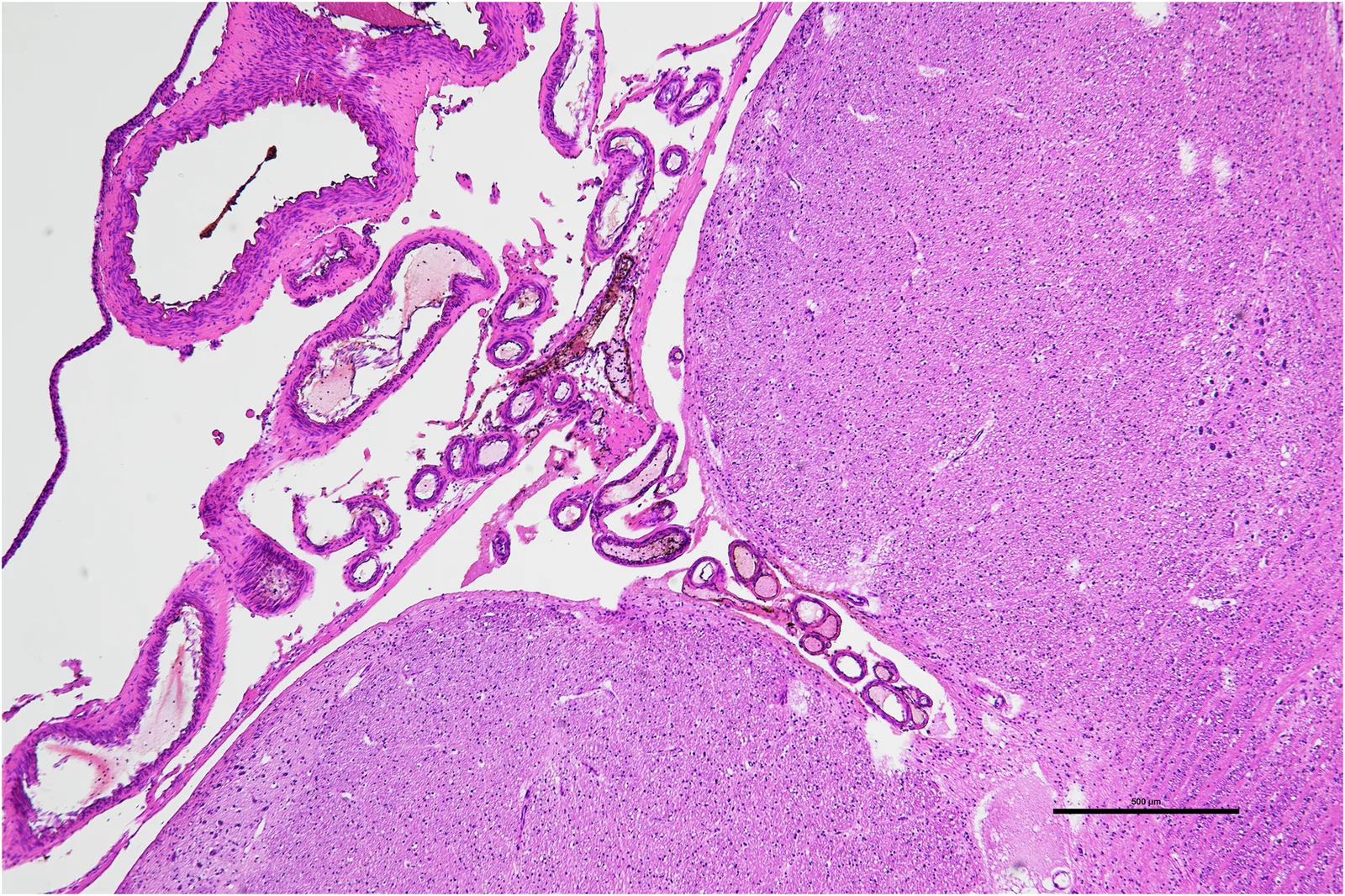
Show a transverse section of the medulla oblongata. Arcuate nuclei are visible, and they appear thin, hypoplastic, and asymmetrical.

**Figure 3:**
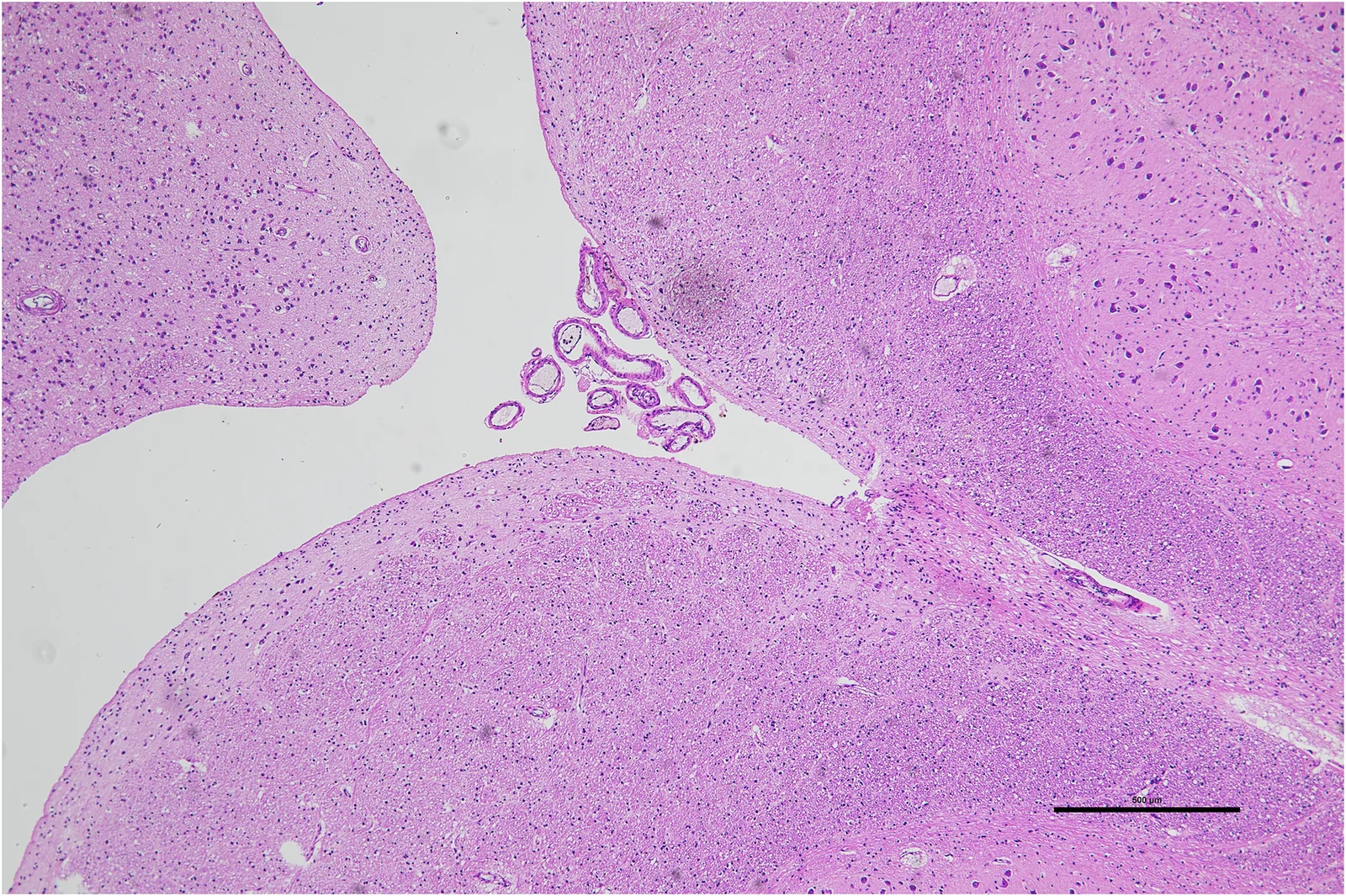
Show a section of the medulla oblongata in which arcuate nuclei appear hypoplastic.

**Figure 4:**
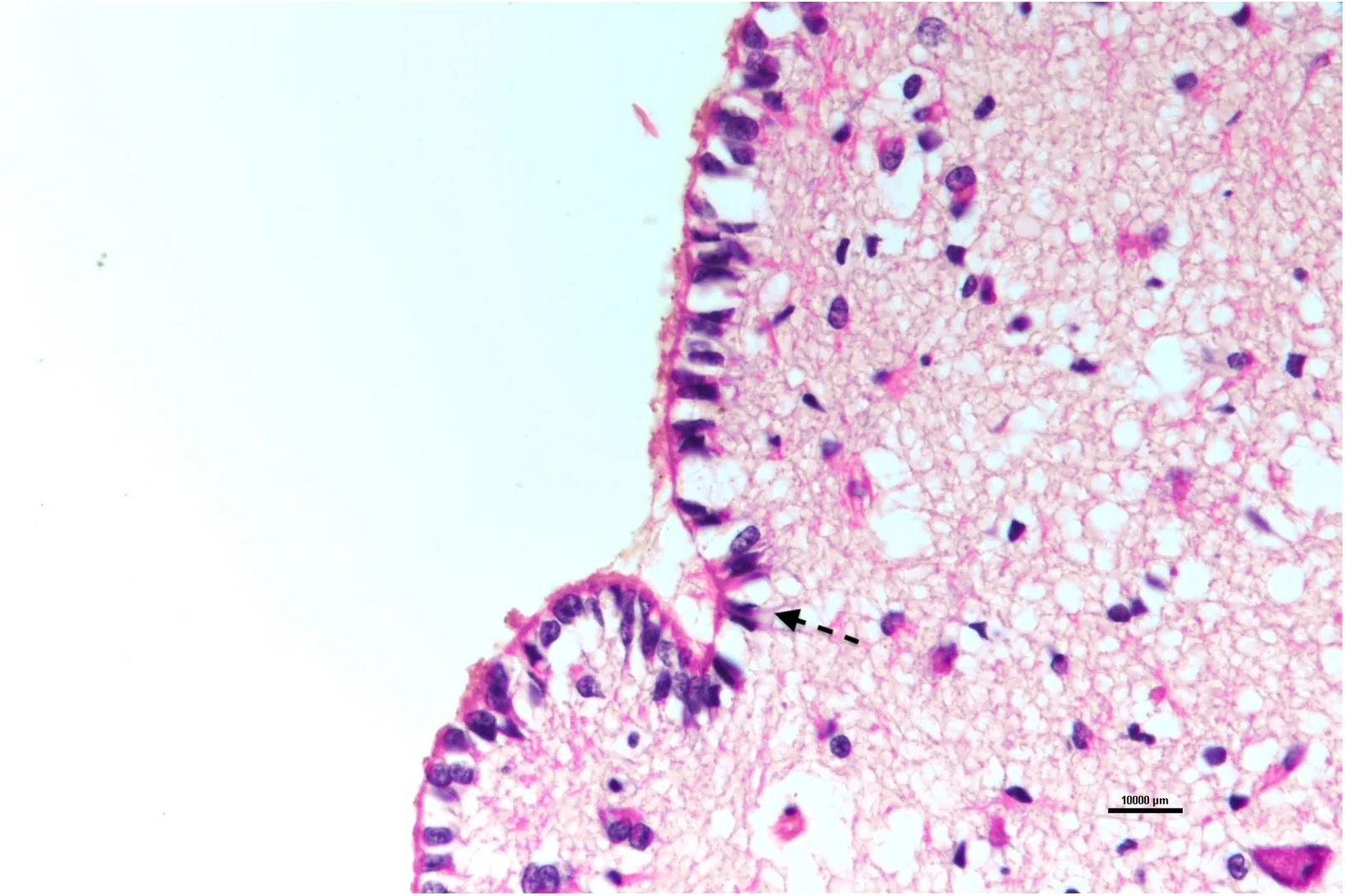
Shows a transverse section of the medulla oblongata single-layered ependyma of the floor of the 4th ventricle that appears not uniformly continuous, with focal presence of tanycytes (black arrow), indicative of delayed ependymal maturation.

A 1-month-old female infant was brought to the Emergency Department by her parents after being found unresponsive several hours following breastfeeding. On admission, the infant was in cardiac arrest and did not respond to advanced life support. A complete medico-legal autopsy was performed. Gross examination revealed nonspecific findings, including subepicardial petechiae, bilateral pulmonary contusions with widespread subpleural haemorrhagic microfoci, and diffuse intra-alveolar haemorrhage. Neuropathological examination demonstrated bilateral asymmetric hypoplasia of the arcuate nucleus (Figures 2–4). In the infant, cocaine and benzoylecgonine were detected in venous blood. Hair analysis revealed the presence of cocaine, cocaethylene, benzoylecgonine, ketamine, and THC Table 2. Segmental hair findings were consistent with prenatal exposure to cocaine and suggested additional postnatal exposure, plausibly mediated through breast milk. Medical records of the pregnancy described unexplained intrauterine growth restriction during the third trimester.

**Table 2:** Summarizes the toxicological results of the analysis performed on two matrices, blood and hair, collected during the autopsy.

Substance	Peripheral blood (ng/mL)	Hair (ng/mg)
Cocaine	13	717
Cocaethylene	Negative (LOD 1 ng/mL)	0,16
Benzoylecgonine	90	39
Ketamine	Negative (LOD 1 ng/mL)	0,76
THC	Negative (LOD 1 ng/mL)	1,8

Toxicological analysis documented maternal use of cocaine and cannabis.

## 4 Discussion

Cocaine use during pregnancy is associated with a wide spectrum of obstetric and neonatal complications, including placental abruption, intrauterine growth restriction (IUGR), preterm birth, and fetal death (12–16). These adverse outcomes are largely mediated by cocaine-induced vasoconstriction and impairment of uteroplacental blood flow, which reduce oxygen and nutrient delivery to the fetus (5, 17, 18). Furthermore, the cocaine-related increase in circulating catecholamines can cause an increase in fetal metabolism, thereby depleting the nutrient reserves in the fetus and further compromising somatic growth (5). Notably, the pregnancy in the present case was characterized by unexplained IUGR in the third trimester, consistent with these pathophysiological mechanisms.

Postnatally, infants may continue to be exposed to cocaine through several routes, including breastfeeding, passive inhalation of crack cocaine, environmental contamination, or accidental ingestion. Among these, breastfeeding represents a potentially significant but under-investigated route of exposure, as cocaine is known to cross the blood-milk barrier and may be ingested repeatedly by the nursing infant (19, 20).

A critical factor complicating the forensic interpretation of cocaine-related infant deaths is the marked difference in cocaine pharmacokinetics between adults and neonates. In adults, the elimination half-life of cocaine is approximately 48 min after intravenous administration, with rapid hydrolysis mediated primarily by hepatic carboxylesterases and plasma butyrylcholinesterase (9). In neonates and young infants, however, both hepatic esterase activity and butyrylcholinesterase levels are substantially lower, resulting in a prolonged elimination half-life. Dempsey et al. reported a cocaine half-life of approximately 11.6 h in a newborn, with a benzoylecgonine half-life of 16 h during the first day of life (21). These pharmacokinetic differences imply that even a single exposure event may produce sustained drug levels and prolonged toxic effects in infants, whereas the same amount would be rapidly cleared in an adult. Furthermore, the specific routes of neonatal exposure and distinct body composition exacerbate this vulnerability. Cocaine efficiently crosses both the placenta and the blood-milk barrier; notably, the milk-to-blood ratio for cocaine has been reported to average 7.8, indicating substantial accumulation in breast milk (22). The lower body weight, higher total body water content, and reduced metabolic capacity of neonates further amplify the effective dose per kilogram. As a result, concentrations that might be considered subtherapeutic or merely incidental in adults are potentially capable of producing severe or fatal toxicity in infants (23). Collectively, these developmental pharmacokinetic disparities firmly underscore the inappropriateness of directly extrapolating adult lethal concentration thresholds to the pediatric population.

Cocaine toxicity is mediated primarily through sodium channel blockade and monoamine reuptake inhibition, resulting in prominent sympathomimetic effects such as vasoconstriction, ischemia, and cardiac arrhythmias, further exacerbated by endothelial dysfunction (24–26). These cardiovascular effects are particularly concerning in newborns and young infants, whose compensatory reserves are limited and in whom even modest distresses in perfusion or rhythm may be fatal. In line with this, Mehta et al., reported diastolic filling abnormalities and possible early structural cardiac involvement in newborns exposed to cocaine *in utero*, suggesting that prenatal exposure may produce subclinical myocardial dysfunction (27).

Cocaine exposure can also have an impact on respiratory function. Inhalation of free-base cocaine, known as crack, induces bronchoconstriction, likely via a topical irritant effect of the cocaine itself or its adulterants (28).

In fetuses and infants exposed during gestation, cocaine-induced alterations of central nervous system development may disrupt the brain circuits that regulate arousal in response to internal stimuli such as hypoxia or hypercapnia, thereby increasing the risk that the infant fails to wake from an apneic episode (29). Pneumocardiographic studies have confirmed, in term newborns exposed to cocaine, a longer duration of apneas and more frequent episodes of bradycardia compared with non-exposed controls (30). These findings provide a biologically plausible link between prenatal cocaine exposure and an elevated risk of sudden unexpected collapse during sleep.

While epidemiological evidence suggests an association between prenatal cocaine exposure and an increased risk of SIDS, the independent contribution of cocaine *per se* remains uncertain. The seminal studies by Durand et al. and Bauchner et al. reported significantly elevated SIDS risk among infants with *in utero* cocaine exposure (4, 5), yet subsequent investigations have yielded inconsistent results. A large meta-analysis by Makarious et al. encompassing over 4.2 million infants confirmed a significantly increased risk of SIDS among those prenatally exposed to illicit substances, independent of socioeconomic factors; however, the risk appeared greater with opioid exposure than with cocaine, and the independent cocaine-specific effect could not be reliably isolated (7). The concurrent use of opioids, cannabis, and tobacco is pervasive among cocaine-using mothers and each of these substances independently elevates SIDS risk, constituting the most important confounders. Socioeconomic deprivation, inadequate prenatal care, maternal mental health comorbidities, environmental tobacco smoke exposure, and unsafe sleep practices are additional variables that modify the cocaine-SIDS risk relationship but are inconsistently controlled for across studies (31). Kandall et al. demonstrated that maternal substance abuse of any kind was associated with subsequent SIDS in offspring, but the contribution of individual substances could not be disentangled (8). The result is a body of literature in which the magnitude of the cocaine-specific effect is difficult to estimate with precision, and in which the reported associations may partly reflect the cumulative impact of multiple co-occurring risk factors rather than the direct pharmacological action of cocaine on the developing infant.

Neuropathological investigations in sudden unexplained perinatal and infant deaths (SUIDs) have repeatedly documented structural abnormalities of the brainstem, particularly hypoplasia and delayed maturation of the arcuate nucleus, a key center involved in respiratory and thermal regulation. Quantitative studies report hypoplasia of the arcuate nucleus in more than 50% of SUID/SIDS cases, supporting its role as a critical vulnerability marker. Structural abnormalities, including neuronal hypoplasia and reduced neuronal density, have been consistently reported in both fetal and infant sudden deaths (32–36).

According to the San Diego definition, SUID encompasses all sudden and unexpected deaths of infants under one year of age, including those that remain unexplained after comprehensive investigation and those eventually attributed to a specific cause. SIDS itself is defined as the sudden and unexpected death of an apparently healthy infant, typically during sleep, which remains unexplained after detailed scene investigation, full autopsy, and radiological, toxicological, and microbiological analyses. The diagnosis of SIDS is therefore one of exclusion and requires the elimination of natural causes, such as congenital anomalies, metabolic, genetic, infectious, or inflammatory diseases, as well as non-natural causes, including abuse, neglect, intoxication, and exposure to toxic or illicit substances (37).

In some jurisdictions, namely in Italy, specific medico-legal protocols, such as Ministerial Decree 07/10/2014, stipulate that the presence of illicit substances generally precludes a diagnosis of SUID/SIDS. This normative framework underscores the weight given to toxicological findings but may inadvertently oversimplify complex cases in which exposure is documented yet the pathophysiological role of the detected substance remains uncertain (38). Another critical legislative point of view was established by the Recommendation no. R (99) 3 of the Council of Europe Committee of Ministers that mandates that a medico-legal autopsy must be performed in all cases of sudden, unexpected death, including SIDS. Specifically, the Recommendation classifies cases with nil or minimal findings into a category that requires an extensive and more comprehensive investigative scheme. In the presence of suspected cocaine exposure, this involves not only the exclusion of poisoning through rigorous toxicology but also a meticulous pathological evaluation (39).

Current systems for detecting and managing cocaine use during pregnancy are subject to significant limitations that contribute to the evidentiary gap discussed above. Maternal substance use screening during antenatal care remains inconsistent across jurisdictions, and the sensitivity of screening tools is variable (40). Stigma, fear of legal consequences, and mistrust of healthcare providers are well-documented barriers that deter women from disclosing substance use, leading to systematic under-detection of cocaine use during pregnancy (41). The present case illustrates this challenge: cocaine use was not identified until post-mortem toxicological analysis, well after the opportunity for clinical intervention had passed.

Cocaine-specific treatment programs are scarce relative to the infrastructure available for opioid agonist therapy, leaving clinicians with limited pharmacological options for managing cocaine dependence in pregnancy (42). This therapeutic gap is particularly significant in low- and middle-income countries, where healthcare resources are already constrained and where cocaine production and use are geographically concentrated. From a forensic perspective, medico-legal protocols that automatically exclude a SUID/SIDS diagnosis whenever illicit substances are detected may oversimplify complex cases in which cocaine acts as a contributing factor within a broader multifactorial vulnerability rather than as the sole proximate cause of death. As Bauchner and Zuckerman observed, the relationship between cocaine exposure and SIDS requires careful contextualization rather than reflexive attribution (43). These systemic gaps, spanning clinical detection, therapeutic management, and forensic classification, collectively contribute to the evidentiary void that the proposed integrated diagnostic protocol in the present manuscript aims to address.

The systematic review conducted for this study identified multiple reports of fetal and infant deaths associated with maternal cocaine use, alongside cohorts of neonates with documented exposure who survived the perinatal period. Across these studies, fetal and infant blood concentrations of cocaine and benzoylecgonine spanned a wide range, with mean fetal blood levels around 0.26 µg/mL and neonatal concentrations extending from tens of µg/L up to 4.8 mg/L in whole blood (44–46). Importantly, similar or overlapping concentration ranges were observed in non-fatal cases, including surviving neonates evaluated within the first week of life (21, 47, 48). When these pediatric data are contrasted with the substantially higher mean blood concentrations reported in adult fatal intoxications, approximately 19,100 ng/mL in acute overdose and 6,200 ng/mL in chronic or binge use, the absence of a clear separation between “fatal” and “survivable” levels in infants becomes evident (10). This pronounced variability, together with methodological heterogeneity among laboratories and potential post-mortem redistribution, severely limits the possibility of establishing validated toxicological cut-off values for diagnosing acute cocaine intoxication as the cause of death in infancy.

Recent non-Western literature on maternal cocaine exposure and infant health outcomes remains limited and does not provide direct epidemiologic evidence on infant mortality. The closest human study identified in our systematic search was a retrospective cohort from La Pampa, Argentina, which reported four-year health trajectories in children prenatally exposed to cocaine and/or cannabis, focusing on developmental outcomes rather than mortality (49). Regional mechanistic studies from Brazil have explored the biological consequences of gestational crack cocaine exposure, including immunological, behavioral, and neurodevelopmental changes, as well as the effects of anti-cocaine vaccination in dams and offspring during the prenatal and lactation periods (16, 50). These studies support the biological plausibility of cocaine-related harm during early development, but they do not substitute for direct epidemiologic evidence on cocaine-attributable infant mortality. The scarcity of non-Western mortality data represents a critical gap and underscores the need for standardized, multicenter surveillance studies in regions where cocaine use is prevalent.

In the present case, the infant’s blood cocaine concentration was low compared with typical adult fatal levels, and autopsy findings comprising of subepicardial petechiae, pulmonary hemorrhages, and arcuate nucleus hypoplasia were non-specific and frequently described in SUID/SIDS. These features exemplify the diagnostic gray zone in which cocaine exposure is unequivocally documented, yet the toxicological data and pathological findings do not allow a confident attribution of death to acute intoxication. Consequently, a rigid application of regulatory criteria that exclude SUID/SIDS whenever illicit substances are present risks misclassifying deaths in which cocaine may have acted as a contributing factor within a broader multi-factorial vulnerability, rather than as the sole proximate cause.

Taken together, our findings and the existing literature indicate that there is currently no consensus on toxicological thresholds that reliably distinguish fatal cocaine intoxication from incidental exposure in infants. This uncertainty has important forensic and public-health implications: it hampers the ability of pathologists to provide definitive opinions in court, complicates surveillance of drug-related infant mortality, and may obscure the true burden of cocaine-associated SUID/SIDS. To address these gaps, systematic collection of paired toxicological, neuropathological, and cardiopulmonary data in all suspected SUID/SIDS cases, including those with low-level cocaine exposure, is essential, ideally using standardized sampling and analytical protocols to facilitate inter-study comparability.

**Figure 5:**
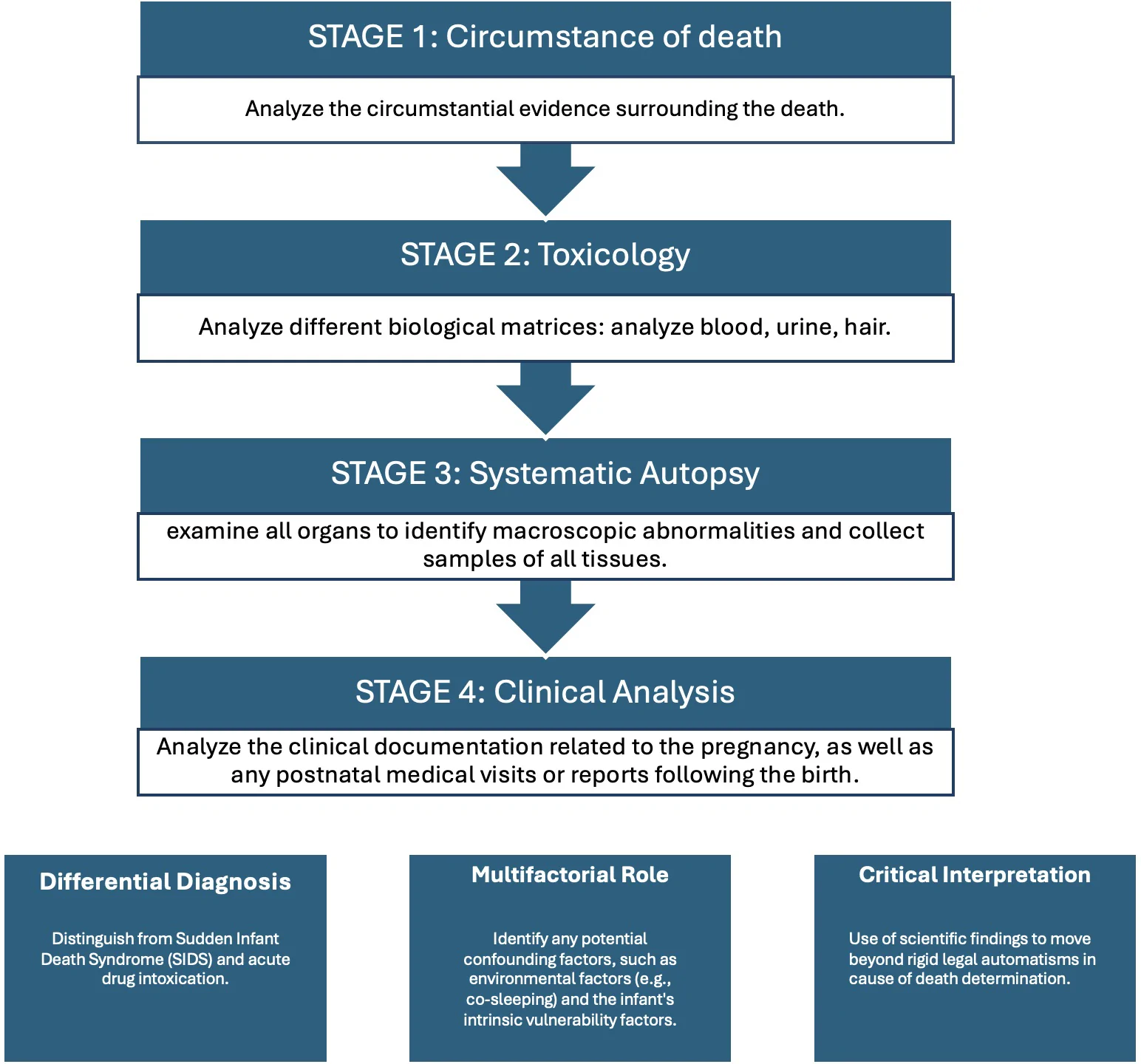
Shows an integrated diagnostic protocol for suspected cocaine-related infant deaths.

The diagnosis of death in infants exposed to cocaine remains a significant challenge due to the absence of validated toxicological thresholds and the presence of confounding factors that frequently overlap with SUID/SIDS. To enhance diagnostic accuracy, we propose a four-stage integrated diagnostic protocol for suspected cocaine-related infant deaths (Figure 5), designed to standardize data collection and support a more nuanced differential diagnosis between SIDS/SUID and acute intoxication.

Given that neonatal blood concentrations can range significantly, isolated blood levels are often insufficient for a definitive diagnosis. Toxicological analysis should comprehend:

- Blood and urine research for both cocaine and its primary metabolite, benzoylecgonine, to identify recent acute exposure.
- Segmental hair analysis to differentiate between chronic *in utero* exposure and more recent postnatal exposure.
- Breast milk research of cocaine, to confirm lactation as a plausible route for ongoing postnatal drug delivery.

The post-mortem examination must go beyond non-specific findings to identify markers of physiological vulnerability and drug-induced injury. Forensic pathologists should rigorously verify external growth parameters to confirm intrauterine growth restriction (IUGR) or general hypotrophy, which are common sequelae of cocaine-induced uteroplacental vasoconstriction. A detailed search for Myocardial Contraction Band Necrosis (CBN) serves as a key histological marker for the catecholamine surge and subsequent ischemia induced by cocaine. Evidence suggests that even prenatal exposure can lead to subclinical myocardial dysfunction and diastolic filling abnormalities. A comprehensive examination of the brainstem is essential, specifically focusing on the arcuate nucleus.

The forensic interpretation must be contextualized within the infant’s clinical and environmental history. A review of obstetric history for unexplained IUGR during the third trimester can support a pattern of chronic gestational exposure. Environmental assessment is necessary to rule out alternative routes of exposure, such as the passive inhalation of crack cocaine smoke or accidental ingestion.

The final phase requires a holistic synthesis of all gathered evidence to move beyond rigid legal classifications. The pathologist must determine if cocaine was the proximate cause of death (acute intoxication) or a contributing factor that exacerbated a pre-existing vulnerability, such as brainstem hypoplasia. In many cases, cocaine acts as a “trigger” within a broader multifactorial framework rather than a sole cause.

### 4.1 Limitations

Several methodological and interpretive limitations must be acknowledged. First of all, hair analysis in infants carries inherent risks of misinterpretation as the neonatal hair has an irregular prenatal growth rate (40). The hair of young children is finer, more porous, and more susceptible to external contamination than that of adults. Cocaine deposited on the hair surface through maternal sweat, sebum, or environmental contact with drug residues can yield positive results that do not reflect systemic exposure. As a recent systematic review and meta-analysis by Cestonaro et al. highlighted, no validated age-specific reference concentrations for cocaine or its metabolites in infant hair currently exist, and the Society of Hair Testing cut-offs established for adults cannot be directly applied to pediatric samples (22). Secondly, the stability of cocaine in biological samples is a well-documented pre-analytical concern. Huertas et al. demonstrated that while cocaine and its metabolites remain stable at −20 °C, they undergo significant degradation at 4 °C, with sodium fluoride preservative substantially retarding hydrolysis (51). The choice of blood collection tube is therefore critical, and the absence of NaF can lead to significant underestimation of ante-mortem cocaine concentrations. Moreover, post-mortem redistribution acts as a further confounding factor. Emaus et al. recently demonstrated site-dependent changes in cocaine and metabolite concentrations between central and peripheral blood compartments, with the direction and magnitude of redistribution varying by sampling site and post-mortem interval (52). The restriction to English-language publications in the systematic review may have introduced language bias, particularly excluding relevant studies from Latin America and other regions where cocaine use is prevalent. Collectively, these limitations underscore the urgent need for standardized pre-analytical protocols and caution forensic pathologists against an over-reliance on any single matrix or concentration value when attributing the exact cause of death in pediatric cases.

## 5 Conclusions

Cocaine abuse remains a pervasive public-health problem affecting over 4.3 million users in the United States in 2024 and 2.7 million young adults in Europe in 2025. Maternal use is still a challenging reality, and it is associated with a markedly increased risk of sudden infant death syndrome and of infant deaths related to cocaine exposure alone. Currently there is no consensus in the literature regarding validated cut-off levels to diagnose acute cocaine intoxication as a cause of death in infants. To resolve these uncertainties, it is essential to implement standardized, systematic collection of paired toxicological, neuropathological, and cardiopulmonary data in every suspected SUID/SIDS case, adopting uniform sampling and analytical protocols to enable inter-study comparability and ultimately to establish evidence-based diagnostic thresholds for this vulnerable population.

## Data Availability

The original contributions presented in the study are included in the article/Supplementary Material, further inquiries can be directed to the corresponding author/s.
